# The Migrant Paradox in Children and the Role of Schools in Reducing Health Disparities: A Cross-Sectional Study of Migrant and Native Children in Beijing, China

**DOI:** 10.1371/journal.pone.0160025

**Published:** 2016-07-26

**Authors:** Ying Ji, Yanling Wang, Lei Sun, Yan Zhang, Chun Chang

**Affiliations:** 1 Department of Social Medicine and Health Education, School of Public Health, Peking University, Beijing, China; 2 Beijing Centers for Disease Control and Prevention, Beijing, China; Örebro University, SWEDEN

## Abstract

Migrants usually exhibit similar or better health outcomes than native-born populations despite facing socioeconomic disadvantages and barriers to healthcare use; this is known as the “migrant paradox.” The migrant paradox among children is highly complex. This study explores whether the migrant paradox exists in the health of internal migrant children in China and the role of schools in reducing children’s health disparities, using a multi-stage stratified cluster sampling method. Participants were 1,641 student and parent pairs from Grades 4, 5, and 6 of eight primary schools in Beijing. The following school types were included: state schools with migrant children comprising over 70% of total children (SMS), private schools with migrant children comprising over 70% (PMS), and state schools with permanent resident children comprising over 70% (SRS). Children were divided into Groups A, B, C or D by the type of school they attended (A and B were drawn from SRSs, C was from SMSs, and D was from PMSs) and whether they were in the migrant population (B, C, and D were, but A was not). Related information was collected through medical examination and questionnaires completed by parents and children. Prevalence of caries, overweight and obesity, poor vision, and self-reported incidence of colds and diarrhea in the previous month were explored as health outcomes. The results partially demonstrated the existence of the migrant paradox and verified the role of schools in lowering health disparities among children; there are theoretical and practical implications for improving the health of migrant children.

## Introduction

Compared with the native population, migrants often have relatively low social status and income level and insufficient access to health services [1, 2], all of which are health risk factors. However, many health indices, such as self-reported health, pregnancy outcomes, and body weight in infants, are better in migrant populations than in native populations [3–5]. This is the “migrant paradox.” This phenomenon and the “healthy migrant effect,” which is used to explain the migrant paradox, have long been the foci of research on the health of international migrants [6]. Similar phenomena have also been observed in internal migration (i.e., migration within a country) [7, 8]. However, some studies in recent years have questioned this interpretation of the data. They have proposed that these observations are linked to the origins of the populations under consideration, as well as demographic characteristics and choices of health indices used in conducting research [9–13].

Children are a special group in migrant populations. On one hand, they live within similar social and family environments as the migrant populations generally, in which low socioeconomic status (SES) generally leads to health disparities between migrant and native children [14–16]. On the other hand, schools can affect migrant children’s health behavior and outcomes [17, 18] and can play an important role in reducing health disparities and improving health equality. Most children spend their time at home and schools. According to Green and Kreuter’s health promotion planning framework [19], environmental contexts might influence children’s health as reinforcing and enhancing factors, such as families and school environments. Schools provide resources to easily intervene in children’s health. Therefore, health disparities in migrant children appear more complex than that of the migrant population in general. However, previous research has mainly examined international migrant children regarding mental health, asthma, obesity, and risky behaviors and addressed the role of family culture, rather than that of schools [5, 20]. In China, migrant children study in various types of schools with various conditions. If the role of schools in children’s health can be demonstrated, settings-based approaches could be used in health interventions in the future.

China presents a highly suitable context for examining the internal migrant paradox. With the rapid development of the Chinese economy, the internal migrant population in China has been growing rapidly. China’s “migrant population” is defined as individuals leaving their residences for cities for a certain period (more than six months) without changing household registration. In 2012, the internal migrant population was approximately 236 million in China [21]. Similarly, the number of children in migrant populations is also increasing. According to 2010 data, 20.8% of the migrant population were children under 14 years old [22], whose living conditions were characterized by instability, overcrowding, poor sanitation, disadvantaged schooling, and social and cultural isolation [23]. In 2010 in Beijing, 28% of children 6–14 years of age were migrants. In other words, when providing health services to children 6–14 years of age, one in every four will be a migrant [24]. However, there have been relatively few studies of the health disparities between migrant and permanent resident children; whether migrant paradox exists among school-age children is also unclear.

To fill this gap in the literature, the present study explores whether the “migrant paradox” exists in migrant children and analyzes the influence of schools and SES on health disparities among children. Therefore, we assume the phenomena might exist among internal migrant children; that is, migrant children are healthier than resident ones and migrant children in schools with better conditions are healthier than those with poor conditions, controlling for SES. In particular, the analysis of the role of schools may provide a reference for schools to contribute to the reduction of health disparities among children.

## Materials and Methods

The study was conducted in April and May 2012 in Beijing. A multi-stage stratified cluster sampling method was adopted. In the present study, “migrant children” refers to those with household registrations outside Beijing who had been living in Beijing for over six months. Among the 16 districts in Beijing, the Haidian and Fengtai districts were chosen due to their relatively large populations of migrant children, comprising 14.6% and 14.06% of all migrant children in Beijing [24]. In each district, schools with medium student sizes (800–1200) were randomly chosen. One state and one private school with migrant children comprising over 70% of total children in the school was chosen in each district (one state migrant children primary school and one private migrant children primary school, i.e., one SMS and one PMS), paired with two state primary schools of similar sizes in which permanent resident children comprised over 70% of total children in the school (state permanent resident children primary schools, i.e., SRS). In Beijing, state schools (SRSs and SMSs) are government-funded under the unified management of the Board of Education; PMSs are funded by private investment and not under the unified management of the Board of Education. SRSs typically have the best conditions among the three school types, with health education courses regularly offered by qualified teachers and basic hygiene amenities (sufficient taps, washrooms, and classroom lights) and a balanced diet provided. SMSs have poorer facilities and are often located in rural-urban fringe zones. PMSs have relatively poor facilities and management conditions, but are preferred by the migrant population due to their low tuition fees and locations within migrant-populated regions. Two classes from Grades 4, 5, and 6 from each included primary school were selected; all students in these classes, as well as their parents, were included as study participants. Overall, 1,641 pairs of students and parents were obtained from eight primary schools, giving a response rate of 92.3%.

Students were divided into four types by their school types and migrant status: Type A represented permanent resident children in SRSs; Type B represented migrant children in SRSs; Type C represented migrant children in SMSs; and Type D represented migrant children in PMSs. Among these, Types B, C, and D all included migrant children, but in varying schooling environments.

The variables of sex, residence duration, and urban/rural registered residency were included in the models as basic demographic variables. Residence duration was a factor influencing migrant paradox [12] and the phenomena may gradually disappear with longer residence duration. Urban/rural registered residency was included as migrant origin types as some researchers hypothesized that the migrant paradox observations were linked to the population origins [9,10]. Age was excluded in the model because participants were from 4–6^th^ grades and the age means among the various groups were not significantly different.

We selected SES to measure families’ environments; it reflected material circumstances as well as educational environment. In addition, many studies had adopted it as a factor reflecting family conditions [25, 26]. In this study, information on family SES was obtained from questionnaires completed by parents; it included the education levels of the children’s parents, average monthly income per person, ownership of durable consumer goods (television, washing machine, refrigerator, air conditioner, computer, or automobile), accommodation type (apartment block, brick bungalow, or adobe bungalow), and whether bathrooms were shared. Because average monthly income per person is a sensitive question, its non-response rate reached 20%. Consequently, in this study, ownership of durable consumer goods was used to replace this variable. Previous research has applied this method [27, 28]. Factor analysis was used in this study, including parents’ education level (no school education, primary school, junior high school or technical middle school, senior high school or technical school, junior college, and university and above were given 1–6 points respectively; the parent with the higher education level was recorded); number of durable consumer goods (television, washing machine, refrigerator, air conditioner, computer, or automobile; the number owned was equal to the mark given, with a maximum of 6 points); and living conditions (consisting of the two variables of accommodation type and whether bathrooms were shared; accommodation type was divided into apartment block, brick bungalow, and adobe bungalow, which were given 3, 2, or 1 points respectively. In China, apartment blocks typically provide better accommodation conditions and reflect better economic status than brick bungalows. In Beijing, a small number of people of very low economic status live in adobe bungalows). Bathrooms being shared or not shared was given 1 or 2 points respectively; this resulted in a maximum of 5 points for living conditions). Variables of these three factors underwent factor analysis to produce SES scores. The construct validity of extraction factors was a = 0.62; the Cronbach’s alpha of the questionnaires was 0.81. The equation was: SES = (0.428 × parents’ education level score + 0.429 × number of durable consumer goods + 0.414 × living conditions score). Factor analysis then transformed SES scores into a standard normal distribution with mean = 0 and standard deviation = 1. Higher scores indicated higher SES.

We selected and measured health indices as follows. Parents reported whether or not children had experienced colds or diarrhea in the past month. Doctors, who received standardized training, conducted medical examinations of the children, including height, weight, caries, and vision. The examination method was based on *Technical Standard for Physical Examination for Students*, published by the People's Republic of China Ministry of Health and the Standardization Administration of China [29]. The five health indices were selected based on common diseases among schoolchildren in China. Most colds and diarrhea were acute communicable diseases; obesity, poor vision, and caries were common chronic diseases regularly checked in physical examinations in state schools in China.

Height was measured as standing body height with 0.1 cm accuracy using a height meter (Model: 2m; Beijing Dong Hua Teng Sports Equipment Co., Ltd). Weight was measured using a leveraged body weight scale with 0.1 kg accuracy (Model: RGT-140; Wuxi Weighing Apparatus Manufacturer).Overweight and obesity were determined by age and sex based on *Body Mass Index reference norm for screening overweight and obesity in Chinese children and adolescents* by the Working Group on Obesity of China [30]. By this standard, a BMI of 18.1 is considered as the threshold of overweight and obesity in 8-year-old children. The thresholds for 9-year-old boys and girls are 18.9 and 19.0 respectively; those for 10-year-old boys and girls are 19.6 and 20.0 respectively; those for 11-year-old boys and girls are 20.3 and 21.1 respectively; those for 12-year-old boys and girls are 21.0 and 21.9 respectively; those for 13-year-old boys and girls are 21.9 and 22.6 respectively. Caries in this study referred to those in both deciduous and permanent teeth. Observation of any one of three types of cases (current caries, filled caries, and lost teeth due to caries) was considered observation of caries. Standard logarithmic distance vision tables were used for vision examination, with naked eye vision < 5.0 defined as “poor vision.” Caries and vision were examined by trained dentists and ophthalmologists.

Each health index underwent classification calculation by the four groups of children. Continuous variables were described using means and standard deviations; analyses of variance were used to compare groups. Categorical variables were described using percentages and chi-square tests were used to compare groups. Each health outcome was analyzed to examine the effect of SES and school type using logistic regression with two models. Model 1 included sex, registered residency, residence duration, and family SES; Model 2 was based on Model 1, but added groupings of children. Group B was of particular interest: Group B children were in the same school type as Group A children, but were in the same household type as Groups C and D children. Group B was therefore used as the control group in the regression analysis of dummy variables. Odds ratios (ORs) and changes in the 95% CI of SES in these two models were used to determine the effects of schools and children’s identities on health indices. Cox and Snell’s R square and the Hosmer-Lemeshow Goodness of Fit index were used to assess model fit. All analyses were conducted using SPSS 13.0 (SPSS Inc., Chicago, IL).

The Institutional Review Board at Peking University approved this study (IRB00001052-12012). Written informed consent was obtained from the participating students and their parents in this study prior to administration of the survey.

## Results

### 1. Basic demographic characteristics

The basic demographic characteristics of study participants are listed in Table 1. The ratio of boys in Group A was lower than those in the other three groups, which is related to the higher male sex ratio among migrant children. There were no significant differences in the average ages among the four groups of children. The ratio of migrant children with rural household registration was highest in Group D (82.6%).

**Table 1:** Basic demographic characteristics of study participants.

	A	B	C	D	χ^2^ / *F*	*P*
	*n* = 650	*n* = 331	*n* = 332	*n* = 328		
Male ratio (*n*, %)	293(45.0)	172 (52.0)	202 (60.9)	184 (56.1)	24.97	<0.001
Average age (years) ($\bar{x}$±*SD*)	10.7±0.94	10.7±1.05	10.7±1.21	10.9±1.18	1.60	0.187
Residence duration ($\bar{x}$±*SD*)	--	9.2±2.54	8.6±2.9	8.6±3.0	16.13	<0.001
Rural registered residency (*n*, %)	38 (5.8)	131 (39.6)	235 (70.8)	271 (82.6)	688.29	<0.001

### 2. Family SES of studied children

Family information of migrant children is listed in Table 2. Parent education levels in Groups C and D were primarily junior high school or below, with the highest ratio in Group D (fathers 87.5%, mothers 89.9%). Parent education levels of Group A children were the highest, with over 50% being university and above. The number of durable consumer goods owned decreased in the order of Groups A, B, C and D, respectively. Groups A and B children mainly lived in apartment blocks, while Groups C and D children mainly lived in bungalows. The rate of sharing of a family bathroom was highest among Group D children (68.1%). SES scores after factor analysis were highest in Group A and lowest in Group D.

**Table 2:** Basic family information of study participants.

		Group A	Group B	Group C	Group D	χ^2^ / *F*	*P* value
Education level of parents (father/mother, %/%)	Primary school and below	0.4/2.5	31.9/10.6	14.3/29.0	26.3/41.1	Father 879.58;Mother 872.28	<0.001
	Junior high school	7.4/9.2	21.0/33.4	55.5/50.3	61.2/48.8		
	Senior high school or technical school	15.0/11.7	17.9/17.0	19.5/14.9	10.1/8.3		
	Junior college	20.6/26.6	29.2/17.3	7.0/4.6	1.5/1.5		
	University and above	56.6/50.1	2.1/21.6	3.7/1.2	0.9/0.3		
Ownership of durable consumer goods (number)	*(*$\bar{x}$±*SD)*	5.7 ±0.65	5.4±0.97	4.4±1.57	3.5±1.70	282.91	<0.001
Type of accommodation (n, %)	Apartment block	616(94.9)	261(78.9)	107(30.5)	39(11.0)	872.13	<0.001
	Brick bungalow	29(4.5)	65(19.6)	219(62.4)	269(76.2)		
	Adobe bungalow	4(0.6)	2(0.6)	13(3.7)	29(8.2)		
	Other	1(0.0)	3(0.9)	12(3.4)	16(4.5)		
Sharing bathroom	(n, %)	46(7.1)	63(19.0)	173(47.9)	244(68.1)	499.19	<0.001
SES score		0.86±0.51	0.38±0.81	-0.71±0.85	-1.33±0.74	801.47	<0.001

### 3. Students’ health disparities and analysis of influencing factors

The health status of students in the four groups is listed in Table 3. Among students, self-reported incidence rate of colds in the past month was not significantly different among the four groups; self-reported incidence rate of diarrhea in the past month and caries prevalence rates were significantly higher among Group D children (29.9% and 33.8%, respectively) than in the other three groups; prevalence rates of overweight or obesity and poor vision were higher among Groups A (31.0% and 71.8% respectively) and B children (31.1% and 64.4% respectively) than those among Groups C and D.

**Table 3:** Health status among various groups (*n*,%).

	Group A	Group B	Group C	Group D	χ^2^	*P* value
Self-reported cold in the past month	243(38.6)	107(33.4)	114(36.1)	118(36.6)	20.45	0.492
Self-reported diarrhea in the past month	80(12.8)	56(17.7)	60(19.3)	93(29.9)	65.52	<0.001
Caries	75(11.5)	40(12.1)	84(25.3)	111(33.8)	89.69	<0.001
Overweight and obesity	198(31.0)	101(31.1)	73(22.7)	67(20.8)	17.00	0.001
Poor vision	467(71.8)	213(64.4)	180(54.2)	126(38.4)	108.99	<0.001

The above health indices underwent logistic regression and results are shown in Table 4.

**Table 4:** Results of logistic regression analysis on influencing factors of health (OR, 95% CI).

		SES	A	C	D	Cox & Snell	Hosmer-Lemeshow
			(B group as reference)			R Square	Good Fit (χ^2^, *p*)
Cold	Model 1	0.92 (0.82, 1.05)				0.007	6.459, 0.596
	Model 2	0.95 (0.81, 1.11)	1.14 (0.84, 1.55)	1.20 (0.82, 1.75)	1.20 (0.78, 1.84)	0.008	4.571, 0.712
Diarrhea	Model 1	0.76 (0.65, 0.88)***				0.023	8.633, 0.374
	Model 2	0.81 (0.67, 0.99)*	0.77 (0.52, 1.16)	0.89 (0.56, 1.41)	1.22 (0.74, 2.01)	0.026	7.019, 0.535
Caries	Model 1	0.77 (0.67, 0.89)***				0.043	21.355, 0.003
	Model 2	1.00 (0.83, 1.20)	1.08 (0.70, 1.68)	2.00 (1.25, 3.20)**	3.04 (1.83, 5.06)***	0.056	16.122, 0.041
Overweight	Model 1	1.26 (1.09, 1.45)**				0.044	6.232, 0.621
and obesity	Model 2	1.04 (0.87, 1.25)	1.11 (0.80, 1.53)	0.60 (0.40, 0.91)*	0.51 (0.31, 0.82)**	0.051	2.878, 0.896
Poor vision	Model 1	1.44 (1.28, 1.63)***				0.059	1.521, 0.992
	Model 2	1.22 (1.04, 1.43)*	1.31 (0.96, 1.78)	0.96 (0.67, 1.39)	0.53 (0.35, 0.81)**	0.070	3.527, 0.897

Model 1 Only SES with adjusted for sex, residence duration, urban/rural registered residency.

Model 2 SES and children’s type with adjusted for sex, residence duration and urban/rural registered residency.

***p<0.001

**p<0.01

*p<0.05.

Results indicated that the incidence of colds in the previous month was not related to family SES or child classification. OR values of SES in Model 2 on the self-reported rate of diarrhea in the previous month was closer to 1; after the variable of child classification was introduced, the effect of SES decreased, even though statistical significance was not detected in this variable.

SES showed a significant effect on caries prevalence in Model 1, with a higher prevalence of caries in families with lower SES scores. However, after introducing the variable child classification (Model 2), the SES effect became insignificant. Compared with Group B children, the OR value of caries among Groups C (OR = 2.00, 95% CI 1.25–3.20) and D children (OR = 3.04, 95% CI 1.83–5.06) was significantly greater.

Increased SES is a risk factor of overweight and obesity. The OR value of SES in Model 1 was 1.26 (95% CI, 1.09–1.45); this value decreased in Model 2 and became insignificant. The OR values of overweight and obesity among Groups C and D children were significantly < 1: children in Groups C and D faced a significantly lower risk of overweight and obesity than Group B children, controlling for SES.

When the prevalence of poor vision was introduced in Model 2, the effect of SES diminished. The OR value of Group D children was 0.53 (95% CI, 0.35–0.81), indicating a lower risk of poor vision.

Regarding the comparison between Groups A and B children, no significance was found in these health variables, indicating that although Groups A and B children had different identities, they had no difference in health indices.

## Discussion

This study’s results support the existence of migrant paradox regarding colds, overweight and obesity, and poor vision among internal migrant children in China, but not regarding diarrhea and caries. Health status in migrant children appears closely related to low family SES. Schools importantly affect health promotion by providing a health-supportive environment. Children studying in state primary schools with better conditions faced a lower risk of some diseases after family SES was controlled. These results partly support our assumption.

The migrant paradox refers to the contrasting observations that immigrants usually experience similar or better health outcomes than native-born populations despite socioeconomic disadvantages and barriers to healthcare use [9]. Researchers testing this phenomenon originally compared migrants to those remaining in the country of emigration, but it is now often applied in the explanation of favorable health outcomes among migrants relative to native-born individuals in host countries. Adding to existing literature, the present study compared the health of migrant children to permanent resident children in China.

Sampling migrant children is difficult since most of them are unregistered. In our study, we sampled them by various types of schools and dropout children were excluded. The Chinese Sixth Population Census in 2010 showed that among 6–11-year-old migrant children, the percentage of children out of schools was 3.52%. Therefore, we believed our sample was representative. SES can be measured by several dimensions, such as occupation, income, accommodation type, and educational level, but various studies adopted all or most of these dimensions [27, 28, 31]. We ignored the occupation dimension in our study because there are not accepted occupational socioeconomic status evaluation and assignment methods yet in China, which would decrease the precision of SES.

In the general description of health indices, the rate of self-reported colds in the previous month showed no statistically significant difference among the groups; the rate of diarrhea, prevalence of caries, rate of overweight and obesity, and rate of poor vision showed statistically significant differences between at least two of the four groups. Among them, diarrhea incidence rate and caries prevalence were highest among Group D children; prevalence of overweight and obesity and prevalence of poor vision were highest among Groups A and B children. Logistic regression analysis showed that, after adjusting for sex, registered residency, residence duration, and family SES, Groups A and B children had no statistically significant difference in any index; Groups B and C children showed statistically significant differences in caries and overweight and obesity; and Group D children had significant differences in caries, overweight and obesity, and poor vision compared with Group B children.

The present findings are valuable in that there have been relatively few studies on migrant paradox in children’s health among internal migrant studies, which are more focused on adolescent and adult populations, with a small number on the health of infants. For example, self-reported physical health among migrant adolescents and adults is better than that of the native population, while psychological health is worse than that of the native population [13, 32, 33]; prevalence of infant anemia among migrant populations is higher than among native infants [34]. Therefore, these studies demonstrated migrant paradox does not always occur and can be affected by factors such as the choice of indices and migrant population characteristics. This is related to the fact that SES and migrants’ identities are distal determinants of health; intermediate variables, such as health behaviors and health services, are factors that determine health directly. The results of this study only partially demonstrate the existence of migrant paradox. First, self-reported incidence of colds in the previous month proved the existence of the paradox among migrant children. Although poor living conditions and low SES increase the chance of respiratory tract infections [35], the self-reported incidence of colds among migrant children was not significantly higher than among permanent resident children, which is a manifestation of migrant paradox. Second, the prevalence of overweight and obesity and prevalence of poor vision among migrant children were lower than those in permanent resident children, which appears to demonstrate the migrant paradox phenomenon, and is also consistent with studies on adolescent health among international migrants [5, 36, 37]. However, the phenomenon of better health than native children is consistent with the relatively low SES of migrant children. In different developmental stages, SES has different relationships with child obesity [38]. In developed countries, epidemiological studies have repeatedly shown that obesity levels are higher in children of the lowest SES [39–41]. Although China is at a stage of low overweight and obesity risk in children with low SES [42], some trends already indicate that the prevalence rate of overweight and obesity in children with low SES is increasing very rapidly [43, 44]. Third, incidence of diarrhea and prevalence of caries cannot prove the existence of migrant paradox, but are consistent with the low SES of migrant children and the conclusions of previous studies that showed high incidence of diarrhea [45, 46] and poor dental health [47, 48] in low SES populations.

The results of this study also indicate that there are differences in health status among migrant children in different types of schools. That is, after adjusting for sex, registered residency, residence duration and family SES, prevalence rates of caries, overweight and obesity, and poor vision in Groups B, C, and D were still significantly different, while no difference was detected between Groups A and B, which shared type of school. Differences in school types represent differences in school conditions and are characteristic of Beijing schools. SRSs attended by Groups A and B children have the best conditions. These schools may provide annual health checks on all students and feedback to parents on the occurrence of common diseases among children. On the other hand, in comparison with the other two types of schools, students in these schools have higher academic pressure and lack adequate physical exercise and sleeping time. (Results of behavioral surveys in this study showed that the percentages of students who often feel pressured by study in SRSs, SMSs and PMSs were 19.0%, 9.9% and 11.3%, respectively. Chi-squared tests showed significant differences between SRSs and the other two types of schools. The average daily physical exercise time was 1.81±1.23, 2.62±1.63 and 2.18±1.63 hours respectively. Chi-squared tests showed statistically significant differences in paired comparisons. The percentages of students who were able to sleep before 22:00 were 50.6%, 73.6% and 75.2%, respectively. Chi-squared tests showed significant differences between SRSs and the other two types of schools). All these are risk factors of overweight and obesity as well as poor vision. SMSs attended by Group C children have poorer facilities and are often located in rural-urban fringe zones. However, as they are state schools under the unified management of the government, they normally ensure the administration of routine health education classes and heath checks. The PMSs attended by Group D children have the worst conditions among the three types of schools, with fewer studying facilities and lower teaching capacity [49, 50]. Perhaps most critically, this type of school does not conduct routine health checks and therefore cannot monitor or provide feedback on students’ health. Groups C and D did not differ significantly regarding the self-reported prevalence of colds and diarrhea in the previous month, although Group C children received a health check each year. This may reflect the contents of this annual checkup. In state schools, weight, height, teeth, vision, and finger blood are examined. Any abnormalities would result in suggested corrections and treatment and parents would be reminded. However, colds and diarrhea are typically acute and infectious diseases closely related to environmental conditions and individual resistance. That is likely why no significant difference was found between Groups C and D regarding these variables. Therefore, different types of schools can indirectly affect students’ health through students’ behavior and through the availability of health services provided by schools. Previous studies have also indicated that schools play an important role in reducing health disparities among children [17, 51]. The effect of health checks provided by schools on reducing the rate of poor vision has also been long proven [52]. At the same time, differences between schools’ facilities and services can generate new health disparities [18]; students’ health is to some extent affected by students’ attendance, for various reasons, of different types of schools, which also deserves attention in improving children’s health. Marmot has argued that “if the major determinants of health are social, so must be the remedies” [53]. If low prevalence may be achieved in some children, it should be achievable for others, as only social and economic factors vary between children or groups of children; these factors’ effects on children’s health can be managed. Thus, determinants of health should be viewed from the perspective of society and structure. More preventative policies should be implemented to address the underlying determinants of health and health-related behaviors.

One advantage of this study is that it is one of a small number of studies on the migrant paradox among internal migrant children and demonstrated the role of schools in reducing child health disparities. The obtained results suggest health issues that should be emphasized among migrant children, as well as the importance of schools in promoting school-age children’s health. The present findings generally suggest that health promotion projects and health services provided should be tailored to school’s particular characteristics to promote children’s health equity.

Despite these important contributions to existing knowledge, the present study has the following limitations. First, its cross-sectional design impedes inferences of causality; this study could only demonstrate that there are health disparities among migrant children in different types of school after controlling for a series of factors, rather than demonstrating that schools were the cause of health disparities among migrant children. Second, colds and diarrhea were self-reported by guardians of children and were not medically diagnosed, which could introduce some recall bias. Third, the examination results for caries among caries patients included both deciduous and permanent tooth caries, so the prevalence of caries was relatively high. Finally, to simplify the statistical analysis, this study used school type to approximate school conditions. However, the facilities, heath regulations, health education classes, and teaching qualifications were also scored to demonstrate that the condition scores of SRS, SMS, and PMS indeed decreased in this order.

## Conclusion

This study partially demonstrates the existence of migrant paradox among internal migrant children in China. Health status in migrant children is closely related to low family SES. Schools play an important role in health promotion by providing a health-supportive environment. The obtained results indicate that we may reduce health disparities among migrant children, as well as between migrant and permanent resident children, through health education at school.

## Supporting Information

S1 File The database that was analyzed for preparation of this manuscript was provided in this file. (XLS)

## Abbreviations

SES: social economic status
SMS: state migrant children primary school
PMS: private migrant children primary school
SRS: state permanent resident children primary school
